# An Exploration of Pepino (*Solanum muricatum*) Flavor Compounds Using Machine Learning Combined with Metabolomics and Sensory Evaluation

**DOI:** 10.3390/foods11203248

**Published:** 2022-10-18

**Authors:** Zhu Sun, Wenwen Zhao, Yaping Li, Cheng Si, Xuemei Sun, Qiwen Zhong, Shipeng Yang

**Affiliations:** 1 Qinghai Key Laboratory of Vegetable Genetics and Physiology, Agriculture and Forestry Sciences Institute, Qinghai University, Xining 810016, China; 2 Laboratory for Research and Utilization of Germplasm Resources in Qinghai Tibet Plateau, Xining 810016, China; 3 College of Life Sciences, Northwest A & F University, Yangling 712100, China

**Keywords:** fruit flavor, pepino, flavor metabolites, machine learning

## Abstract

Flavor is one of the most important characteristics that directly determines the popularity of a food. Moreover, the flavor of fruits is determined by the interaction of multiple metabolic components. Pepino, an emerging horticultural crop, is popular for its unique melon-like flavor. We analyzed metabolomics data from three different pepino growing regions in Haidong, Wuwei, and Jiuquan and counted the status of sweetness, acidity, flavor, and overall liking ratings of pepino fruit in these three regions by sensory panels. The metabolomics and flavor ratings were also integrated and analyzed using statistical and machine learning models, which in turn predicted the sensory panel ratings of consumers based on the chemical composition of the fruit. The results showed that pepino fruit produced in the Jiuquan region received the highest ratings in sweetness, flavor intensity, and liking, and the results with the highest contribution based on sensory evaluation showed that nucleotides and derivatives, phenolic acids, amino acids and derivatives, saccharides, and alcohols were rated in sweetness (74.40%), acidity (51.57%), flavor (56.41%), and likability (33.73%) dominated. We employed 14 machine learning strategies trained on the discovery samples to accurately predict the outcome of sweetness, sourness, flavor, and liking in the replication samples. The Radial Sigma SVM model predicted with better accuracy than the other machine learning models. Then we used the machine learning models to determine which metabolites influenced both pepino flavor and consumer preference. A total of 27 metabolites most important for pepino flavor attributes to distinguish pepino originating from three regions were screened. Substances such as *N*-acetylhistamine, arginine, and caffeic acid can enhance pepino‘s flavor intensity, and metabolites such as glycerol 3-phosphate, aconitic acid, and sucrose all acted as important variables in explaining the liking preference. While glycolic acid and orthophosphate inhibit sweetness and enhance sourness, sucrose has the opposite effect. Machine learning can identify the types of metabolites that influence fruit flavor by linking metabolomics of fruit with sensory evaluation among consumers, which conduces breeders to incorporate fruit flavor as a trait earlier in the breeding process, making it possible to select and release fruit with more flavor.

## 1. Introduction

Pepino (*Solanum muricatum*) is a genus of eggplant in the family Solanaceae, native to the Andes of South America [1]. In recent years, pepino has gained widespread recognition worldwide, not only for its unique taste and distinctive aroma, but also for its health benefits; its fruits have many benefits, they have been used traditionally as remedy of mellitus diabetic, hypertension, and sprue [2,3]. Unlike most crops of the Solanaceae family, pepino is considered a “false fruit” when other crops are consumed as vegetables, some have argued it is a vegetable-type fruit due to light-flavor and taste, but highly appreciated worldwide not only for its unique taste and distinct flavor also for its health benefits. It was provided that name by the Spaniards because its flavor resembles cucumber when it is unripe [4]. The most commonly used modifier is dulce (sweet), in reference to the sweeter taste of pepinos as compared with cucumbers [5]. In China, pepino is mainly grown in the Jiuquan and Wuwei regions in western Gansu and the Haidong region in Qinghai [6]. Our previous study examined the types and contents of metabolic substances in pepino fruits produced in these three regions by LC-MS [7], but we did not investigate the substances affecting the flavor of pepino fruits. 

The flavors of fruits are not determined by a single dominant compound, but rather by the interaction of multiple metabolic components. As for many fruit species, varieties differ in their flavor and taste, ranging from sourness to sweet such as *Punica granatum* L. [8], *Prunus persica* L. [9], *Cucumis melo* L. [10], etc., with a complicated taste combination. Metabolomics techniques have been used in recent studies to explore metabolites in various plants. Metabolomics is one of the more “distal” of the omics techniques [11]. In addition to technological advancements, the metabolomics field is evolving rapidly [12]. In numerous cash crops, especially of horticultural crops, metabolomics have been identified and characterized because of their agronomic importance. Except for fewer analyses focusing on targeted metabolites (between 1 and 23), untargeted metabolites (between 50 and 200) analysis was used in most studies [13]. Some of the metabolites are well-characterized, but have not been fully investigated in others [14]. One main reason is the lack of clear definition potential untapped in the metabolic network. This technique is only beginning to be applied to biological questions.

A fruit’s flavor is understood to be influenced most by sweetness and acidity [15]. Modern fruit breeding has focused considerable effort on improving yield, shelf life, and disease resistance, while flavor has been relatively neglected [16]. As shown by the decreased sucrose equivalent levels and similar ratios of sucrose equivalents to citric or glutamic acid contents, despite being useful to reduce the incidence of pests and viral diseases, it reduces the organoleptic quality [17,18]. As a result of these studies, we know more about the metabolite composition in relation to fruit flavor. This can contribute significantly to the evaluation of fruit’s nutritional value and freshness. Several factors are responsible for flavor perception in fruit flavor, including sugars and acids. The taste buds on the tongue detect sugars and acids, resulting in the sense of taste. Over the years, many surveys have been conducted on fruits to examine their flavor profile and hundreds of metabolites have been reported from different pathways, especially in the terpenoids and volatile organic compounds [19,20,21,22]. The metabolomics profile of fruits has only been compared with human evaluations in a few of these studies. Since fruit flavor is a complex feature, it is more difficult to determine the correlation between phenotypes and metabolites accurately [23]. However, recently, great effort has been taken predominantly on blueberry flavor by Vincent and Luís Felipe [24,25]. They identified which VOCs both impact blueberry flavor and influence consumer liking by machine learning models. Machine learning is the study of computer algorithms that are automatically improved through experience and can be classified as supervised learning, unsupervised learning, and deep learning depending on the learning method, with a variety of algorithms including Bayes models, Regression Models, Random Forest, Support Vector Machines, and Neural Network applied to different types of data [26,27,28]. A variety of topics have been successfully predicted using machine learning. With big data technologies and high-performance computing, machine learning has opened up new opportunities for multidisciplinary biotechnology research including accurate prediction of protein structures [29], plant–pathogen interactions [30] and image-based plant phenotyping [6,31]. We can explore the relationship between metabolomics data and flavor phenotypes through machine learning, allowing flavor to be incorporated earlier in the breeding process and fruit varieties with more flavor can be selected and released.

Taken together, an LC-MS/MS-based metabolic profiling method was applied to quantify three planting areas of pepino fruit metabolites to explore the reasons why certain territoriality characteristic fruit were preferred over others. A sensory evaluation of pepino fruit from three regions was conducted and based on our previous metabolic analysis data, we evaluated and derived pepino flavor by using machine learning to research suitable pepino flavor prediction models and their main metabolites affecting pepino flavor. Our comprehensive analysis provides new insights into the metabolomics control of pepino flavor and provide a roadmap for future breeding and cultivation efforts to enhance pepino flavor.

## 2. Materials and Methods

### 2.1. Experimental Materials

The pepino used in the sensory analysis was collected from Minhe, Qinghai, China and Jiuquan, Wuwei, Gansu, China, where light-oval fruit (LOF)-type pepino varieties are grown mainly through greenhouses. The metabolomic detection used pepino grown in the same conditions and harvested at the same time. A total of 85 differential metabolites were used for analysis, including amino acids and derivatives, nucleotides and derivatives, organic acids, phenolic acids, lipids, saccharides, and alcohols identified in a total of 18 samples from these three regions [7]. Metabolomics profiling identified metabolites and their relative contents are shown in Table S1.

### 2.2. Methods

#### 2.2.1. Sensory Evaluation of Pepino

The flavor of pepino fruit was assessed by a sensory panel of 123 consumers who evaluated the flavor attributes of sweetness, sourness, flavor intensity, and liking preference of pepino fruit (Table S2). The taste panel consisted of 123 teachers and students and faculty members from Qinghai University, and each member of the panel was informed about the experiment and agreed to conduct it. Pepino from all three regions was cut into small pieces and blended thoroughly in each. They randomly tasted pepino from three selected regions and were asked to rinse their mouths with salt water after tasting the fruit from each region. Scores were based on the general markers of the hedonistic degree scale (gLMS) [32]. The panelists were informed of the scoring procedure prior to the test, and all scores were based on the strongest sensation they had ever felt, ratings from 0 to 100 for all attributes indicate an increase in the intensity of consumer perception of the attribute.

Statistical analysis was performed with the ggstatsplot package (https://github.com/IndrajeetPatil/ggstatsplot, accessed on 13 August 2022). Metabolomics data as well as scores were normalized by R’s scale function (Table S3) for further analysis. The purpose of the assessment was to understand the potential flavor preferences of people in the three regions for pepino fruit.

#### 2.2.2. Data Analysis

We used WGCNA [33] to calculate correlations between metabolites to facilitate our understanding of the relationships between metabolites, and then visualized this metabolite association network through Cytoscape 3.9.1 [34]. For the contribution of metabolites to flavor ratings, we drew on the linear model fitted by Colantonio et al. [25] to estimate the proportion of variation in flavor ratings explained by each metabolite group including amino acids and derivatives, nucleotides and derivatives, phenolic acids, organic acids, lipids, saccharides and alcohols with the aid of the ASReml-R package [35]. The fourteen models include Bayes A, Bayes B, Bayes C, Ridge Regression, Linear Regression, LASSO, Random Forest, Elastic Net, Reproducing Kernel Hilbert Space, Neural Network, Kernel Partial Least Squares, Linear Support Vector Machines, Radial Support Vector Machines, and Radial Sigma Support Vector Machines, which based on conventional machine learning algorithms were used to predict sensory traits. We assessed the predictive ability of each model through a 5-fold cross-validation method. In each fold, 18 sets of metabolic groups and score data from three regions were randomly divided into three groups, each containing 5 data, and 4 of the 5 data were used as the training set, and the remaining one was used as the test set. The prediction accuracy was calculated with reference to the method provided by Colantonio et al. All the above calculations were implemented in R 4.2.1., exploring important metabolites affecting pepino flavor attributes using β coefficients of Bayesian A models and variable importance of Neural Network models. MetaboAnalyst 5.0 [36] generated the PCA scores plot and loading plot, visualized with R-package ggplot2 [37], and graphically enhanced with Adobe Illustrator CC 2019. PCA was used to determine whether the screened important metabolites could distinguish pepino fruit originating from three regions.

## 3. Results

### 3.1. Statistics of Sensory Evaluation

**Figure 1:**
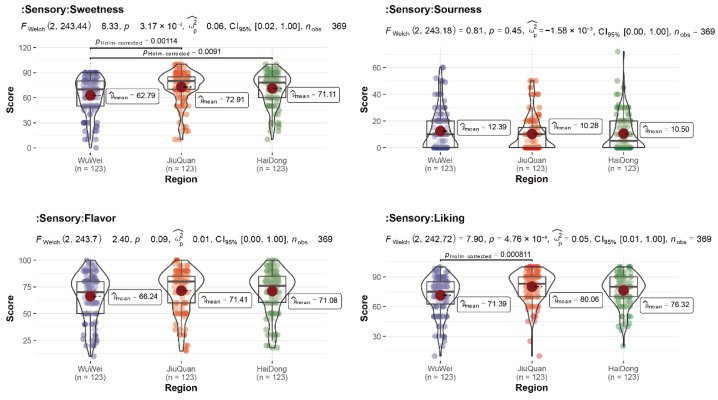
Stats-violin plot of testers’ sensory evaluation of pepino in three regions.

We tallied the assessment ratings of 123 evaluators on the sweetness, sourness, flavor intensity, and liking of pepino fruit from the three locations in order to identify the prospective consumer preferences for pepino from the three regions (Figure 1, Table S2). There was a significant difference in perceived sweetness of pepino fruit between the three regions, Jiuquan (72.91) having the highest sweetness score, followed by Haidong (71.11), and pepino produced in the Wuwei (62.79) region receiving the lowest sweetness score. Jiuquan (*p* = 0.00114), Haidong (*p* = 0.0091), and Wuwei regions reached significant differences in sweetness scores. The liking for pepino from the three regions also differed significantly, with pepino from the Jiuquan region receiving more favorable ratings from testers in the same way as sweetness, and correspondingly, pepino from the Wuwei was perceived less favorable by more people. Although there was no significant difference between the three regions on sourness and flavor intensity, there was a similar scoring trend, Jiuquan pepino scored highest in flavor intensity and lowest in sourness, while Wuwei pepino scored lowest in flavor intensity and lowest in sourness. The Haidong was always in the middle. In all indicators, the testers scored more concentratedly, with only a few scoring lower.

### 3.2. Metabolic Network

**Figure 2:**
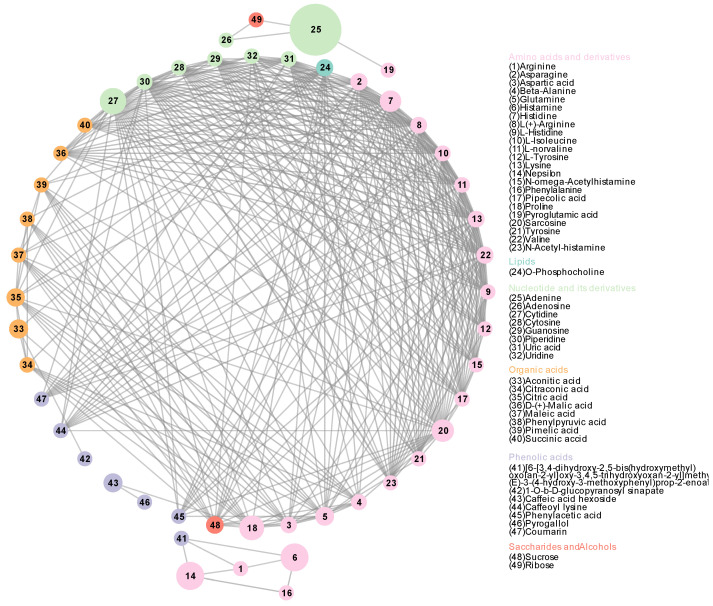
Each metabolite node is sized based on its betweenness centrality. The thickness of the line connecting the metabolites is indicated by the weighted value (Tables S5 and S6). Identification of each metabolite is indicated by a number in the legend.

An analysis of all pepino fruits from three regions was conducted using weighted correlation networks (Figure 2). The results indicate the relationship between metabolites, based on the weighted with threshold value of (>0.3); out of the 85 metabolites, 23 amino acids and derivatives, 1 lipid, 8 nucleotides and derivatives, 8 organic acids, 7 phenolic acids, 2 saccharides and alcohols metabolites make up this network of metabolite relationships, which is largely consistent with the pathway of biosynthesis. For example, amino acids and derivatives, and nucleotides and derivatives as precursors for the synthesis of metabolites in the organism, have the most connections with the rest of the metabolites throughout this metabolic network, suggesting that amino acids and derivatives, and nucleotides and derivatives play an important role in pepino metabolism, affecting phenolic acids, organic acids, lipids, saccharides, and alcohols substance accumulated in the pepino. Moreover, the presence of sucrose was closely related to organic acids. Histamine (6), Nepsilon (14), proline (18), sarcosine (20), adenine (25), cytidine (27), aconitic acid (33), citric acid (35), and caffeic acid hexoside (43) are present as large nodes in the metabolism-related network, indicating that these metabolites are important relational hubs in the metabolic network, closely linking individual metabolites.

### 3.3. Contribution of Various Metabolites to Flavor Perception

**Figure 3:**
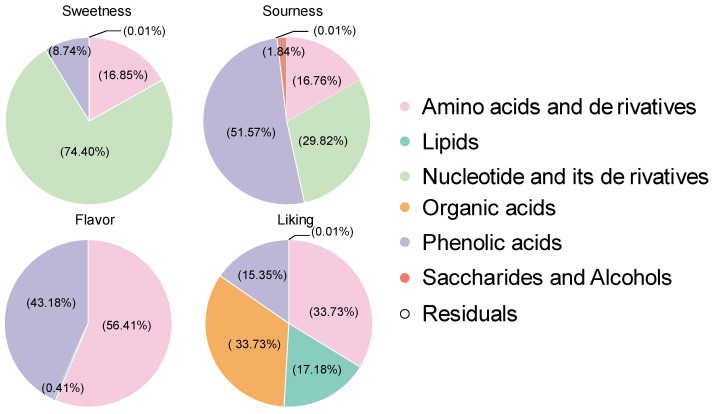
Contribution of metabolite groups to sensory characteristics.

The fruit metabolome was partitioned into modules according to their biochemical classifications to determine if variation in consumer sensory panel ratings can be explained by metabolites. After that, each module was analyzed individually to determine how it explained the variance in consumer sensory perception. Metabolites grouping accounted for a large proportion of variance, whereas residuals explained little. The sweetness of pepino was mainly explained by nucleotides and its derivatives explaining 74.40% of the variation, while the remaining 16.85% and 8.74% of the variation was explained by amino acids and derivatives, and phenolic acids, respectively. The variation in pepino acidity was mainly explained by phenolic acids (51.57%). Moreover, amino acids and derivatives (16.76%), and nucleotides and derivatives (29.82%) also played a large role. Saccharides and alcohols also explained 1.84% of the variation. Amino acids and derivatives (56.41%) and phenolic acids (43.18%) alone explained 99.59% of the variation in flavor intensity in pepino fruit, with the remaining 0.41% provided by lipids. The liking level of consumer preference for pepino fruit required a combination of metabolites, with organic acids, lipids, amino acids and derivatives, and phenolic acids explaining 33.73%, 17.18%, 33.73%, and 15.35% of the variation, respectively (Figure 3).

### 3.4. Consumer Flavor Preference Prediction

**Figure 4:**
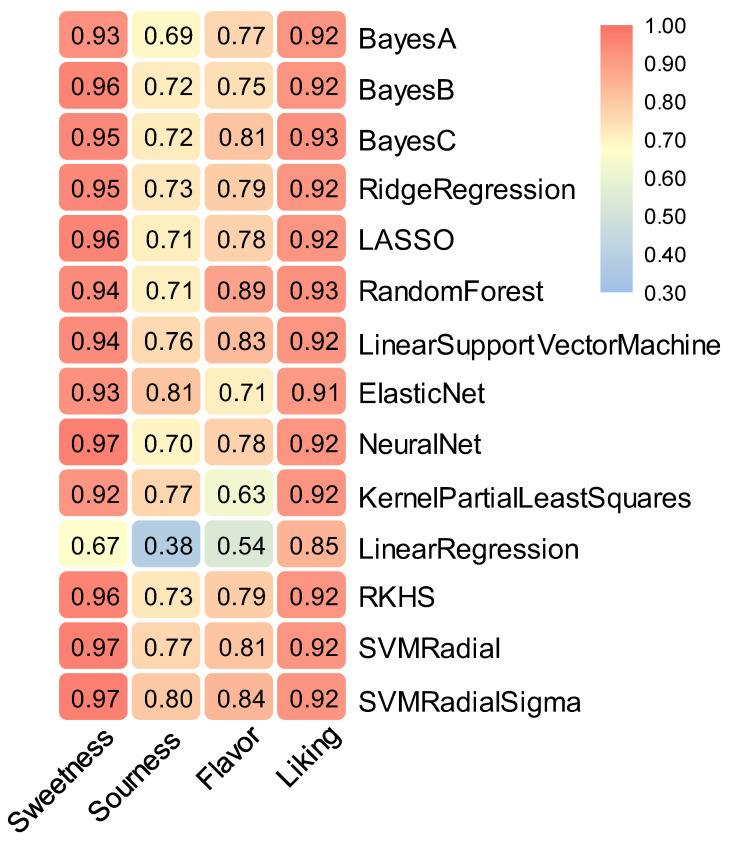
Model prediction of sensory characteristics.

To predict the sensory characteristics of various metabolites, we explored the relationship between metabolites and sensory characteristics by using 14 statistical and machine learning methods. All validation metrics were calculated on the predictions from the 5-fold cross-validation. The mean of the five cross-validations was the final accuracy value of the predictions. The results showed that the 14 statistical and machine learning methods were more accurate in predicting the pepino sweetness and liking preference, and the prediction accuracy of all methods was above 0.92 except for the Linear Regression method. For sweetness, the SVM and Neural Net model with the highest prediction accuracy improved the prediction accuracy by 44.8% compared with the Linear Regression model with the lowest prediction accuracy. Moreover, the Random Forest and Bayes C models with the highest prediction accuracy for liking improved by 9.4% compared with the Linear Regression model with the lowest prediction accuracy. As for the two sensory characteristics of sourness and flavor intensity, not all methods were applicable, and the prediction accuracy ranged from 0.38 to 0.89. The Elastic Net model had the highest prediction accuracy of 0.81 for sourness, while the Random Forest model provided the highest prediction accuracy of 0.89 for flavor intensity. Meanwhile, the Linear Regression model predicted the pepino sourness and flavor intensity with an accuracy of only 0.38 and 0.54, respectively. Overall, the more accurate model for predicting all sensory features is the SVM Radia Sigma, with prediction accuracy above 0.80 for all features and 0.97, 0.80, 0.84, and 0.92 for sweetness, sourness, flavor intensity, and liking, respectively (Figure 4).

### 3.5. Important Metabolites Associated with Sensory Characteristics

**Figure 5:**
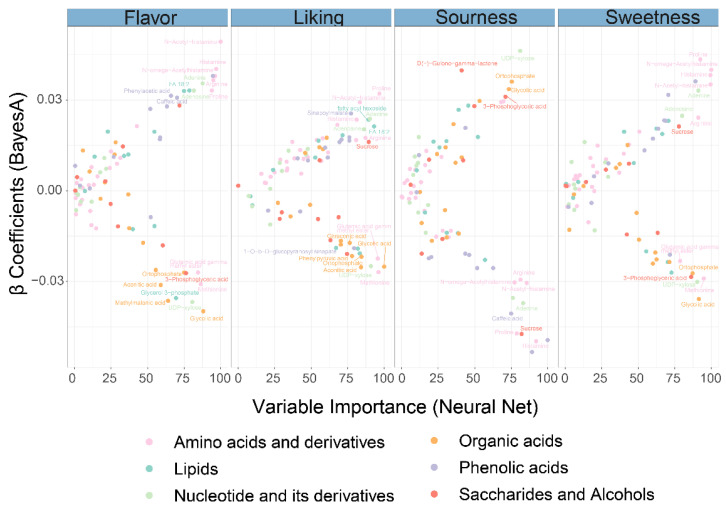
Role of metabolites on sensory panel. β coefficient from the Bayes A (*X*-axis), variable importance from the Neural Net (*Y*-axis).

Using Bayes A and Neural Networks, we calculated the correlation between metabolites and sensory characteristics in order to understand how they affect sensory perception. The β coefficient indicates whether sensory characteristics have been enhanced or suppressed. On the other hand, the Variable Importance of Neural Net explains the degree of effect provided by a metabolite on the flavor profile as a result of the joint action with other metabolites, and the value of Variable Importance from 0 to 100 corresponds to an enhanced effect. The graphs (Figure 5) show metabolites with β Coefficients >0.3 or <−0.3 and Variable Importance >75. For pepino flavor strength, amino acids and their derivatives including N-Acetyl-histamine, histamine, N-omega-acetylhistamine, arginine, proline, two phenolic acids including caffeic acid and phenylacetic acid, nucleotides and derivatives such as adenine and adenosine, and FA 18:2 in lipids all play an important role and have a positive enhancement effect on pepino flavor intensity. In contrast, organic acids such as glycolic acid, orthophosphate, methylmalonic acid, aconitic acid, and other metabolites such as Methionine, UDP-xylose, and glycerol 3-phosphate had a reverse inhibitory effect on flavor intensity. The liking of consumer preference for Pepino fruit was mediated by a combination of metabolites, and metabolites such as Methionine, glycerol 3-phosphate, UDP-xylose, aconitic acid, phenylacetic acid, and sucrose all acted as important variables in explaining the liking preference. Organic acids such as glycolic acid, orthophosphate, and sucrose appeared as positive enhancing and negative inhibiting variables in sourness perception, respectively. Conversely, for sweetness enhancement and reduction, sucrose and organic acids such as glycolic acid and orthophosphate acted as significant contributors.

### 3.6. Metabolites Distinguish Pepino Regional Origin

**Figure 6:**
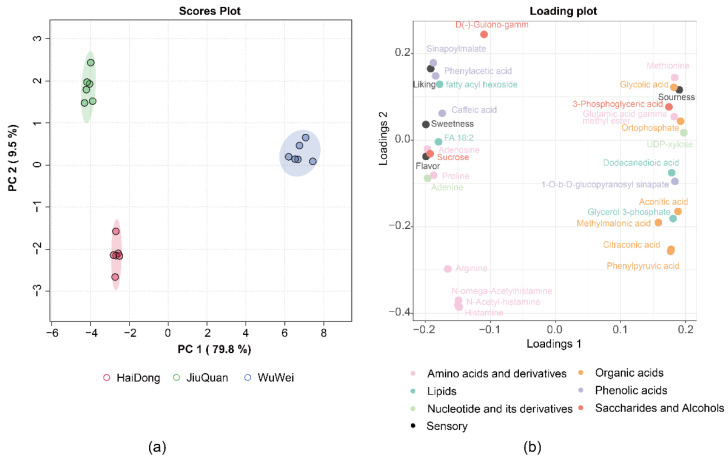
PCA plots of the scores (**a**) and loadings (**b**) of the three regional pepino.

We selected 27 metabolites of high importance for consumer sensory characteristics, which were used as important metabolites for PCA analysis, and we wanted to investigate whether we could distinguish pepino fruit from the three regions by these substances. Based on the results of the PCA analysis (Figure 6a) we found that the 18 samples were divided into three groups according to their regional origin, the first two components of PCA explained 40.6% and 21.8% of the variance, respectively. Consumer perceptions of sweetness, liking, and flavor intensity were located on the left side of the plot, and were closer to phenolic acids, lipids, and saccharides and alcohols including sucrose, indicating that these substances were more influential on consumer perceptions of sweetness, liking, and flavor intensity of pepino fruit. The sourness of pepino fruit appeared on the right side and was more closely related to organic acids such as glycolic acid, orthophosphate, and amino acids and its derivatives (Figure 6b, Table S6).

## 4. Discussion

Flavor is one of the most important characteristics that directly determines the popularity of a food. The most frequently reported flavor category was fruit (high school: 66.1% (95% CI, 62.4–69.5%)) [38]. Plant breeders have traditionally focused on improving producers’ traits such as yield, while neglecting consumer-oriented traits such as flavor. Fruit quality is an integrative combination of several metabolites. An important human selection trait is the flavor of fruits which is primarily a balance between sugars and acids [39]. Different cultivars produce fruits with diverse sugar compositions which contribute to a distinctive flavor profile, although studies in this area lack examination of the relationship between incredibly complex metabolites and difficult appraised flavor [40,41]. Several recent studies have demonstrated that high-throughput metabolomics and flavor-evaluative integration analysis using statistical and machine learning models were able to predict consumer sensory panel ratings based on a fruit’s chemistry [24,25]. It is a new and elegant approach that means flavor and nutritional alterations can be corrected with precision molecular breeding, especially for non-model species. Pepino is one of neglected and underutilized horticultural crop growing widely on the Loess Plateau, China; here, we analyzed the metabolomic data of the three different pepino growing areas belonging to Haidong, Wuwei, and Jiuquan from south to north on the west of the plateau. The purpose of this study was to investigate the flavor phenotype in a group to select three different pepino growing areas that can be distinguished and were preferred by consumers.

The contributions of sugars, acids, and volatiles in the minority of Solanaceae crops flavor have been well-characterized, but those of other metabolites detected by LC-MS have not been as well-investigated. Pepinos are considered orphan crops because of low genetic resources, and exotic and intense flavors. Early pepino metabolic has not been completely investigated despite improvements in metabolomics technologies. Currently, there are only two studies on the literature which used HS-GC-MS to determine volatile compounds in pepino [42,43]. Among these approaches, metabolite detection is frequently used to understand volatiles metabolic status, making a direct link between phenotypes and genotypes. As a result of these data, pre-existing aroma were identified, and an important group of volatile compound was characterized. The mechanism by which these volatiles modulate our taste preferences remains unclear, despite their importance and are limited due to no high-throughput metabolomics data being available for pepino. In this work, we developed three different pepino growing areas of fruit metabolomics data that addressed the above problems. Application of the LC-MS-based metabolic profiling method has been a routine way to analyze how continuous dependent variables interact with independent variables, such as environment, plays a significant role in specialized metabolite accumulation [44]. There are numerous conceptual parallels between metabolites and biological species, which make it possible to ask questions about flavor-related metabolites through statistical analysis, and machine learning in particular.

There is no doubt that these technologies are poised to transform food metabolomics research in the near future. As a main hypothesis, we focused explicitly on flavor notes, a trait systematically observed by pepino customers. Three identical pieces of pepino were rated in three different contextual settings for sweetness, sourness, flavor, and overall liking. Variations exist in the metabolite properties of the same pepino varieties grown in different regions. This context may be agricultural management or influenced by the environment [45,46,47]. This is clear from the sensory evaluation analysis of variation in three different pepino growing areas. Consumers rated the pepino fruits from Jiuquan to have the greatest (*p* < 0.01) sweetness, flavor, and linking, while the Haidong rated similar (*p* < 0.01) to Jiuquan. Clearly, pepino fruits from Jiuquan were greeted more enthusiastically. In our previous study, soil nutrients were used as an environmental factors analysis which showed that total nitrogen (TN), total phosphorus (TP), available phosphorus (AP), available potassium (AK), and organic matter (OM) altered the metabolite profile in both Jiuquan, Haidong, and Wuwei of pepino fruits [7]. It is not difficult to explain this observation. Soil nutrients significantly altered the metabolite profile of pepino fruits, which in turn altered consumer preferences. This is a complex and systemic issue because fruit flavors are not influenced by a single dominant compound but rather by a multitude of volatile components interacting together. These questions were at the center of our study: What kind of metabolites are responsible for flavors perception of pepino [44]? The highest contribution results according to sensory evaluation showed that nucleotide and its derivatives, phenolic acids, amino acids and derivatives, saccharides, and alcohols were dominant in the sweetness (74.40%), sourness (51.57%), flavor (56.41%), and liking (33.73), respectively. It is believed that nucleotides and their derivatives have umami, which enhances the sweet and mellow taste of food [48,49]. In addition, the overall distribution of nucleotides and their derivatives was similar when taken in three areas. In fruits, although most sensory studies have not explicitly addressed taste perception in their sensory panels, there is evidence suggesting that adenine, arginine, and proline contribute to “umami”, “floral”, and “fruity” taste notes [50,51,52]. These nucleosides are biologically important, but their effects on the sensory quality of fermented products were smaller than those that resulted from their phosphorylated forms and other metabolites we found [53]. Additionally, a variable importance analysis was performed for significant metabolites identified in the aforementioned sensory analyses. Where indicated, sucrose includes contribution from sweetness, sourness, and linking. Undoubtedly, Consumer acceptance of pepino fruits were expected to contain more sweetness because pepino is recommended for diabetic and sugar-free diets due to its low sugar content [54,55]. However, Sensory evaluation of pepino is based on general population judgment of evaluation criteria, which is subjective.

There is little consensus about what constitutes an ideal snack, but the most common items are sweetened fruits [56]. Only some sweet fruits produce long-term memory [57]. The main targets of current fruit breeding programs are weight, acidity, sweetness, firmness, and polyphenol content, as do other horticultural crops including pepino [54,58]. The information provided by this study is essential new information for pepino breeders who must rethink breeding strategies to increase diversity of sweetness. More directly, sensory diversity studies can help us to adjust our strategies for evaluation and breeding. In this article, we argue that metabolites analysis can provide a more accurate assessment of taste and consequently flavor. Unfortunately, plant breeding is currently restricted to incremental improvements in quantitative traits that often depend on the selection of rare and naturally occurring mutations in gene-regulatory regions. However, new editing technologies allow us to make genetic edits very efficiently to achieve big data-assisted targeted breeding [59,60]. A similar approach has been used in other contexts in the plant literature and applied directly to fruit sweetness enhancement. Some recent examples are reported in *Vitis vinifera* L. [61], *Fragaria vesca* [62], and *Zea mays*. L [63]. It has also been shown that the CRISPR/Cas9 gene-editing system is capable of creating heritable targeted mutations in transgene free plants [64]. A. tumefaciens has been injected into aboveground meristems and used to improve plant regeneration recently [65,66]. This is advantageous in the case that CRISPR/Cas9-based editing for the fine-tuning sugar content using the above genome editing and transformation regeneration system strategy is adopted as the fast and effective for pepino.

In addition to plant breeding, metabolomics can also be used in genetics and food science research to predict flavor characteristics. To our knowledge, few studies have identified baseline metabolomic predictors since model calibration requires many sensory panels and comparison of different statistical methods, particularly machine learning. However, the most advanced research concerning metabolomic selection for enhanced fruit flavor has been performed on the blueberry [24,25]. Despite its widespread potential, the continuing success of flavor phenotyping depends upon an adequate supply of metabolomics data. An analysis of the test accuracies obtained from supervised machine learning algorithms showed that the machine learning phases significantly influenced the accuracies [67]. Our work included the evaluation of a range of statistical and machine learning models for the prediction of flavor quality based on metabolite information. Finally, we employed 14 machine learning strategies trained on the discovery samples to accurately predict the outcome of sweetness, sourness, flavor, and liking in the replication samples. It is evident that the majority of the sweetness and liking exhibit similar behavior in both models except for Linear Regression analysis. The results of a multivariate Linear Regression analysis can be skewed by a small sample size [68]. As a result, the Radial Sigma SVM model performed better than other machine learning models. Through linear or nonlinear kernel functions (Radial SVM), SVM is a supervised learning classification algorithm that constructs a hyperplane in a higher dimensional space [69]. Across all tasks, we found that overall prediction accuracy was high. However, we wanted to quantify prediction accuracy separately for each task. However, in consideration of the practical solution and application of calculation procedure, a Neural Net model was selected. The reasons may be that some algorithms may have some disadvantages in our study, such as Bayes A, linear support vector machine, and kernel partial least squares. Compared with the other algorithms aforementioned, this can be due to more iteration rounds, a large amount of training data, K-mean selection or unstable results, and possible interference between variables [70].

Using Bayes A and Neural Network models, 27 metabolites closely related to consumer sensory characteristics were identified. We found that the result of PCA loadings of these 27 metabolites were similar to those predicted by the model when analyzing PCA data, with phenolic acids such as caffeic acid, lipids such as FA18:2, and sucrose having a positive effect on consumers’ sensory characteristics including sweetness, liking, and flavor intensity. Correspondingly, there was an inverse inhibition of sourness. For sourness, despite the enhancement of sourness in the pepino fruit by organic acids such as glycolic acid and orthophosphate, other sensory characteristics of the fruit are diminished in consumer perception. As a result, there is a high degree of confidence in Bayes A and Neural Network models in predicting pepino flavor. Accordingly, we speculate that caffeic acid, FA18:2, sucrose, glycolic acid, and orthophosphate are important substances that affect the flavor of pepino fruit. However, we know little about the effect of substances other than sucrose on fruit flavor, so more in-depth research is needed.

The present study also has several limitations. Furthermore, for some metabolites, although we observed high prediction accuracy in the machine learning models, their response in the real world might not reach the expectations. Cultivation fosters plant naturalization by reducing environmental stochasticity [71]. However, the complexity becomes high, and metabolic assays in vitro do not capture the complexity of environment or all the biological context in the same way as simulation and prediction methods. In addition, most statistical methods used in compound selection assume that metabolites are being sampled independently, which is certainly not true given the intricate correlations and connections seen among metabolites in metabolic networks [72].

## 5. Conclusions

Several conclusions can be drawn based on the experimental results. Consumers generally prefer pepino grown in Jiuquan. These sensory characteristics are mainly influenced by nucleotides and derivatives, phenolic acids, amino acids and derivatives, and saccharides and alcohols such as sweetness, acidity, flavor intensity, and liking. A total of 14 machine learning models were tested for their ability to predict consumer preferences, with the Radial Sigma SVM model showing the best results. A screening of metabolites affecting pepino’s flavor revealed that caffeic acid, FA18:2, sucrose, glycolic acid, and orthophosphate were able to influence four sensory characteristics of consumers, and distinguish between pepino fruits from three different regions. As a result of our study, it appears that machine learning is capable of detecting pepino flavor substances accurately. However, the generic recommendations for one algorithm cannot be made due to the differences in research environments and subjects. There may be cases in the future where more algorithms are used to develop models to make appropriate development of desirable flavors from a particular study.

## Data Availability

Data is contained within the article.

## Supplementary Materials

The following supporting information can be downloaded at: https://www.mdpi.com/article/10.3390/foods11203248/s1, Table S1: Metabolites and their relative contents identified by metabolomic analysis in pepino from three regions; Table S2: Sensory evaluation of the pepino from three regions of by a consumer sensory panel of 123 members; Table S3: Normalized metabolomic data and sensory scores; Table S4: Betweenness centrality, classification, and number of nodes in metabolic networks; Table S5: Weight values of connections between metabolites in the metabolic network; Table S6: Loading value of metabolites in PCA analysis of pepino from three regions using 27 important metabolites screened.
